# Cost of peer mystery shopping to increase cultural competency in community clinics offering HIV/STI testing to young men who have sex with men: results from the get connected trial

**DOI:** 10.1186/s13561-023-00447-6

**Published:** 2023-06-02

**Authors:** Victoria L. Phillips, Ashley Xue, Marné Castillo, Dalia Santiago, Taylor Wimbly, Lisa B. Hightow-Weidman, Rob Stephenson, José A. Bauermeister

**Affiliations:** 1 Rollins School of Public Health of Emory University, 1518 Clifton Road, Room 614, Atlanta, GA 30322 USA; 2 Division of Adolescent Medicine, The Children’s Hospital of Philadelphia, Philadelphia, USA; 3 Department of Pediatrics-Retrovirology, Baylor College of Medicine, Texas Children’s Hospital, Dallas, USA; 4 Prevention Research Center, Morehouse School of Medicine, Atlanta, USA; 5 Institute of Global Health and Infectious Diseases, University of North Carolina, Chapel Hill, USA; 6 School of Nursing and the Center for Sexuality and Health Disparities, University of Michigan, Ann Arbor, USA; 7 School of Nursing, University of Pennsylvania, Philadelphia, USA

**Keywords:** Costs, Activity-based costing, Cultural competency, Quality improvement, Young men who have sex with men, HIV-testing

## Abstract

**Introduction:**

Cultural competency has been identified as a barrier to lesbian, gay, bisexual and transgender (LGBT) populations seeking care. Mystery shopping has been widely employed in the formal health care sector as a quality improvement (QI) tool to address specific client needs. The approach has had limited use in community-based organizations due in part to lack of knowledge and resource requirement concerns. Several mystery shopping initiatives are now being implemented which focus on the LGBT population with the goal of reducing barriers to accessing care. One subset targets men who have sex with men (MSM) to increase uptake of human immunodeficiency virus (HIV) testing. No study investigates the costs of these initiatives. Get Connected was a randomized control trial with the objective of increasing uptake of HIV-prevention services among young men who have sex with men (YMSM) through use of a resource-locator application (App). The initial phase of the trial employed peer-led mystery shopping to identify culturally competent HIV testing sites for inclusion in the App. The second phase of the trial randomized YMSM to test the efficacy of the App. Our objective was to determine the resource inputs and costs of peer-led mystery shopping to identify clinics for inclusion in the App as costs would be critical in informing possible adoption by organizations and sustainability of this model.

**Methods:**

Through consultation with study staff, we created a resource inventory for undertaking the community-based, peer-led mystery shopping program. We used activity-based costing to price each of the inputs. We classified inputs as start-up and those for on-going implementation. We calculated costs for each category, total costs and cost per mystery shopper visit for the four-month trial and annually to reflect standard budgeting periods for data collected from September of 2019 through September of 2020.

**Results:**

Recruitment and training of peer mystery shoppers were the most expensive tasks. Average start-up costs were $10,001 (SD $39.8). Four-month average implementation costs per visit were $228 (SD $1.97). Average annual implementation costs per visit were 33% lower at $151 (SD $5.60).

**Conclusions:**

Peer-led, mystery shopping of HIV-testing sites is feasible, and is likely affordable for medium to large public health departments.

## Introduction

While HIV incidence has decreased in the US population, it has remained constant or increased among men who have sex with men (MSM), particularly among younger age groups [1]. Improving rates of HIV testing for this demographic is an essential component of the national plan to prevent cases of HIV and achieve the Centers for Disease Control and Prevention’s (CDC) goal for ending the HIV epidemic [2].

Extensive research and intervention efforts have been aimed at increasing test uptake among young men who have sex with men (YMSM). Lack of cultural competency among providers has been identified as a significant barrier to care engagement for this group [3]. Studies have begun to explore ways to eliminate cultural barriers in the wider lesbian, gay, bisexual and transgender (LGBT) population, such as through peer navigation to promote use of PrEP among transgender women and MSM [4–6]. Improving cultural competency for YMSM has yet to be systematically addressed [3].

Public health department quality improvement (QI) has been increasingly promoted to improve service delivery, efficiency and responsiveness to public needs [7]. The National Association of County and City Health Officials now offers a step-by-step guide, while recognizing that QI efforts must be actionable within organizational budgets [8]. Studies have also evaluated pilot public health department projects in select states [9]. One tool yet to be implemented widely is mystery shopping.

Mystery shopping, derived from business marketing, is a tool used in a wide range of settings to evaluate and monitor service performance, and inform QI initiatives to increase customer satisfaction [10]. Mystery shopper visits can range from low-intensity phone calls to high-intensity interventions, such as in-person visits, with findings recorded and reported in near real-time. These visits can provide data for QI regardless of form or setting [11].

The price of mystery shopping is determined by the content and mode of the visit. Costs to undertake mystery shopping include the start-up costs to initiate the program, such as script creation and training in its execution [12]. On-going implementation costs, after the initial investment, reflect the length and type of encounter, the number of encounters per provider, the complexity of the investigation scenario and post visit reporting arrangements [13].

In the formal health care arena, mystery shopping has been used to improve patient experiences in areas such as appointment referral, lab testing, receipt of mammograms, pharmacy delivery, physician waiting rooms and in family planning clinics [14–18].

Recent work in the US and internationally has begun to explore the feasibility of using mystery shopping as a QI tool in community-based HIV-testing sites. Several studies have investigated the level of cultural competency and the nature of provider/client interactions at community-based organizations with the goal of improving elements, such as site friendliness and provider knowledge, as a means to increase test uptake among the YMSM [19–21]. A guide for conducting mystery shopping within HIV community-based clinics has also recently been published [22]. However, hesitancy regarding its use exists among community-based organizations due to lack of knowledge about implementation requirements and costs.

The Get Connected trial focused on YMSM at-risk of contracting HIV and employed a QI intervention. The trial objective was to increase uptake of HIV-prevention services in this group through use of a resource-locator App populated with LGBT-friendly clinics. The initial phase of the trial identified culturally competent HIV testing sites for inclusion in the App. The second phase of the trial randomized YMSM to test the efficacy of the App.

In the first phase, high-intensity, peer-led mystery shopping was conducted in clinics recruited from three cities with high-HIV incidence: Atlanta, Houston, and Philadelphia, serving as study sites. Clinics determined to be YMSM-friendly HIV testing sites, based on visits, were included as locations in the mobile App [23]. Research centers in cooperation with public health departments served as implementation hubs to proxy medium to large public health departments or other community-based organizations which might sponsor these initiatives.

The objective of this paper is to investigate the financial sustainability and resource requirements for possible adoption of the mystery shopping model. We documented the resource inputs and used activity-based costing to estimate start-up, implementation and total costs, along with cost per shopper visit, for four-months, an arbitrary period determined by the trial, and annually to reflect expenses over a standard budgeting period [23].

## Methods

We focused on non-research-related tasks. Based on input from site staff, we created an inventory of activities, materials and equipment required to develop and implement mystery shopping. We used program budgets and activity-based costing to determine program costs for the period September 2019 through September 2020 [24, 25].

Staff reported hours spent on each activity. Labor costs were calculated based on reported hours spent on activities, multiplied by the median hourly wage for each staff category. Activities that were handled centrally due to RCT site responsibilities, such as clinic identification, were allocated equally across sites. Cost values for materials were drawn from program budgets. Peer shoppers visited each clinic twice and were paid $50 per visit, including de-briefing.

For each site we calculated start-up costs or those incurred prior to initiation of mystery shopping which cannot be recouped. We also calculated implementation costs which become a recurrent budget expense if the program is adopted [26]. We then calculated total costs, the sum of the two. We averaged all values the sites.

Using published data on visit numbers, we calculated implementation and total cost per mystery shopper visit for the four-month duration of the trial. We then estimated annual costs by adding eight-month on-going implementation costs, consisting of visit coordination and visits, to the four-month values. We estimated annual cost per shopper visit by assuming the rate of average visits for the four-month period applied through the remainder of the year [27]. Data are available from the author upon request.

## Results

Mystery shopping activities, inputs and their associated costs were allocated to start-up and implementation, shown in Table 1. Data are provided for each site in order of size, measured in terms of number of shoppers, and averaged across sites for the trial period. Shopper protocol development, training staff to educate peer shoppers in protocol execution and program management constituted start-up activities. The first two were handled by one site as part of the trial design and thus show little variation. Average four-month start-up cost per site was $10,001 ($39.8 SD) with project management constituting 58% of the total.

**Table 1:** Activities and Costs of Per-Led Mystery Shopping by Study Sites

Activities and Costs	Atlanta ($)	Dallas ($)	Philadelphia ($)	Mean ($) (SD)	Percent of total (%)
Start-up Activities					
Project initiation management	5779	5873	5804	5819 (39.76)	58
Shopper protocol development^1^	3284	3284	3284	3284 (n/a)	33
Training research site staff training in shopping protocol	898	898	898	898 (n/a)	9
Subtotal: start-up costs	9961	10,055	9986	10,001 (39.76)	100
Implementation Activities					
Clinic identification^1^	466	466	466	466 (n/a)	5
Recruitment of Mystery shoppers	2529	2824	3862	3072 (571.90)	31
Training of mystery shoppers	843	811	1216	957 (183.87)	10
Incentives for mystery shopper training	600	900	900	800 (141.42)	8
Miscellaneous costs for training (food, parking)	101	134	44	93 (37.03)	< 1
Coordinating mystery shopper visits to sites	1111	1048	1816	1325 (348.28)	13
Mystery shopping visit incentives	1600	2000	4500	2700 (1283.23)	27
Staff debriefing with mystery shoppers on site visit findings	578	441	728	582 (117.33)	6
Subtotal: implementation costs	7828	8623	13,533	9995 (2522.84)	100
Total cost	17,789	18,678	23,519	19,995 (2517.77)	100
Sites					
Number of sites (n)	17	19	30	22 (5.72)	
Number of visits (n)	34	38	60	44 (11.43)	
Costs per four- month trial duration					
Implementation cost per visit	230	227	226	228 (1.97)	
Total cost by visit	523	492	392	469 (55.91)	
Cost per year					
Implementation cost per visit^2^	145	150	159	151 (5.60)	
Total cost per visit	248	233	214	232 (13.88)	

^1^ Handled centrally by University of Pennsylvania and allocated across all three sites

^2^ Clinic identification, visit coordination, clinic visits, and debriefing constituted on-going implementation activities

Implementation began with identifying clinics to serve as shopping sites. This also handled centrally by one site. With the cooperation of the local departments of public health, study staff used AIDSVu.org and Google to collect clinic data. Elements included: information on location, services offered, opening hours and contact information. Costs for clinic identification totaled $1398 of which one-third, $466, was allocated to each site.

Other implementation activities included peer recruitment and training of shoppers and coordinating and executing shopper visits, which together constituted two-thirds of four-month average implementation costs of $9995 (SD $2522.8). Costs generally increased with site size with Philadelphia, having the highest costs due to increased costs of shopper recruitment and visit coordination.

Average total four-month costs were $19,995 (SD $2517.8) and were evenly split in percentage terms between start-up and implementation. Implementation costs per visit averaged $228 (SD $2.00) and decreased with site size as did total costs per visit at $469 (SD $55.9), reflecting the costs of increasing visit numbers.

Annual costs are shown at the bottom of Table 1. Average annual total costs, the sum of the initial start-up costs and the estimated 12-month implementation costs, produced an average total of $20,142 (SD $10,297). Average annual implementation cost per visit was $151 (SD $5.6) with an average annual total cost per visit of $232 (SD $13.88).

## Discussion

We identified the activities and resources required to undertake high-intensity, peer-conducted mystery shopping programs to increase cultural competency among community-based clinics offering HIV testing. Our findings indicate that instituting peer mystery shopping to increase HIV-testing among YMSM is feasible given that public health department staff are highly likely to be equipped to undertake mystery shopping activities. Programs were run centrally at study sites in three areas to model conditions for medium to large public health department or community-based organizations. 

Based on the cost of resource inputs, annual average cost per visit was approximately $150. Costs declined with site size, reflecting economies of scale whereby costs decline as start-up costs are spread over an increasing number of visits. Costs varied significantly based on the duration of the program, with costs decreasing as the implementation period increased from four months to one year. Annual values are most relevant for public health departments and community-based organizations, given standard annual budgeting periods. Average total costs fell by over-half from the four-month period to the annual period, while average implementation costs fell by a third, primarily as visit numbers increased, both demonstrating the economies of scale of the mystery shopping programs.

Start-up and implementation costs are important to separate as one indicates the initial investment requirement and the other the on-going cost of the program once it has started. Average start-up costs were around $10,000 per site. These costs appear minimal in comparison with the size of many public health department budgets. For example, in Georgia, location of the Atlanta site, the current state public health department proposed budget for fiscal year 2022, was $298 million dollars [28]. Given its relative costs, mystery shopping may be reasonably integrated into many public health departments, particularly urban areas, as part of their overall QI efforts and expenses.

Average annual implementation cost per visit of $151 (SD $5.6) were at the lower end of published estimates ($50 - $5000) [9]. The intervention evaluated here likely provides an upper bound on mystery shopping costs. The Get Connected visit protocol required two, high-intensity visits per clinic whereas community adoption may only require one, be of lower intensity and have fewer reporting requirements.

Costs presented here reflect the context of the Get Connected trial, conducted in high incidence urban setting and thus may not be generalizable to dissimilar locations. Some values may also have been overestimated. Staff were asked to identify and report time spent on activities related to undertaking mystery shopping only and not research-related costs.

The difficulty of separating these two, particularly in terms of project management hours, may have led overestimation of costs. Salaries to value time reflect those of academic research settings and staff had to have skills related to research in addition to those required to conduct mystery shopping. Thus, wage rates may be higher than those for community staff not engaging in research.

Costs generally increased with site size, with Philadelphia having higher costs in terms of recruitment and training of peer shoppers, coordinating shopper visits, and shopper payments. It should be noted, however, that Philadelphia region was the first site within the clinical trial. As a result, its increased costs may also be linked to unexpected costs associated with being the first site brought on-line.

Other assumptions may have led to an underestimation of costs. We assumed no additional equipment was involved as the App was loaded onto mobile phones already in use by peer shoppers. Reported material costs were minimal reflecting that the intervention relied on mobile devices. No costs were included for acquisition of the App given it is available through open-source software. Annual implementation costs did not account for staff turnover, as none occurred during the four-month period.

Other possible biases are of unknown direction. Costs reported here also reflect the geographical area and features of the local market under investigation, such as ease of access to shopping targets and of shopper recruitment. Staff hours were based on recall which may have led to an over or under-estimate of costs.

Peer-navigator findings from mystery shopping visits were communicated to clinics with the goal of promoting service modification to reduce access barriers to YMSM. This feedback, however, was not communicated formally nor was the degree to which changes were made. This shortcoming needs to be addressed and efforts expanded to help clinics address the cultural barriers identified and subsequent confirmation that service modifications were made.

In addition, user feedback was initially explored through focus groups that provided input during App development. The trial assessed willingness to use and length of use once the App was deployed. Soliciting end-user feedback, however, could provide important information for continuous quality improvement. Feedback could range from including affirmation that the clinic was LBGT-friendly, such as through a “like”, a user clinic rating or qualitative feedback to the App developers. All of which would likely increase value and promote sustainability.

Considering future adoption, it may be possible to institute a lower intensity protocol to identify LGBT-friendly clinics, such as reducing visits to one, or changing the intensity of the visit. Creation of a peer-led, detailed mystery shopper training toolkit, with sample scenarios included, may reduce start-up costs. In addition, the model could be easily modified to include other groups, such as transgender youth, which would further increase its value.

## Conclusions

Lack of cultural competency in HIV testing clinics is a known barrier to YMSM, and the wider LGBT population, accessing services in the US [4–6]. Implementation of mystery shopping, as a pre-cursor to the Get Connected trial, shows that the approach is feasible and a promising QI tool which can be used to help develop and identify culturally competent HIV/STI testing sites.

Here we investigated the resource and financial requirements to inform possible sustainability for peer-led, high intensity mystery shopping programs aimed at YMSM. Public Health Departments, in high incidence urban area, would be suited to launch such programs. The initiatives appear to be highly affordable and a relatively minor expense for public health department and community-based organizations of this type. However, the approach may not be viable for individual clinics or small public health departments to initiate on their own given centralization is necessary to reap economies of scale or declining average costs per visit.

While the US health care delivery and structure of community-based clinic system is unique, the methodological approach and use of activity-based costing is replicable in other countries.

Given the public health goals of promoting health and responding to community needs, peer-led, mystery shopping offers a promising tool to improve service delivery to an underserved, vulnerable population. Increases in testing can reduce cases of HIV thereby helping achieve the CDC goal of eliminating HIV. In addition, establishing linkage to care can increase the overall health of YMSM.

## Data Availability

The datasets used and/or analysed during the current study are available from the corresponding author on reasonable request.

## List of abbreviations

CDC: Centers for Disease Control and Prevention
HIV: human immunodeficiency virus
LGBT: lesbian/gay/bisexual/transgender
MSM: men who have sex with men
PrEP: post exposure prophylaxis
QI: quality improvement
STI: sexually transmitted infection
YMSM: young men who have sex with men
