# Correction: Cost of peer mystery shopping to increase cultural competency in community clinics offering HIV/STI testing to young men who have sex with men: results from the get connected trial

**DOI:** 10.1186/s13561-023-00453-8

**Published:** 2023-07-26

**Authors:** Victoria L. Phillips, Ashley Xue, Marné Castillo, Dalia Santiago, Taylor Wimbly, Lisa B. Hightow-Weidman, Rob Stephenson, José A. Bauermeister

**Affiliations:** 1 Rollins School of Public Health of Emory University, 1518 Clifton Road, Room 614, Atlanta, GA 30322 USA; 2 Division of Adolescent Medicine, The Children’s Hospital of Philadelphia, Philadelphia, USA; 3 Department of Pediatrics-Retrovirology, Baylor College of Medicine, Texas Children’s Hospital, Dallas, USA; 4 Prevention Research Center, Morehouse School of Medicine, Atlanta, USA; 5 Institute of Global Health and Infectious Diseases, University of North Carolina, Chapel Hill, USA; 6 School of Nursing and the Center for Sexuality and Health Disparities, University of Michigan, Ann Arbor, USA; 7 School of Nursing, University of Pennsylvania, Philadelphia, USA

**Correction**: ***Health Econ Rev*13**, **34 (2023)**

10.1186/s13561-023-00447-6

Following publication of the original article [1], the authors identified an error in the surname of Lisa B. Hightow-Weidman.

The incorrect author name is: Lisa B. Hightow-Wideman.

The correct author name is: Lisa B. Hightow-Weidman.

The author group has been updated above and the original article [1] has been corrected.
